# Global Sensitivity Analysis Based on Entropy: From Differential Entropy to Alternative Measures

**DOI:** 10.3390/e23060778

**Published:** 2021-06-19

**Authors:** Zdeněk Kala

**Affiliations:** Department of Structural Mechanics, Faculty of Civil Engineering, Brno University of Technology, 602 00 Brno, Czech Republic; kala.z@fce.vutbr.cz

**Keywords:** sensitivity analysis, importance measure, uncertainty quantification, entropy measures

## Abstract

Differential entropy can be negative, while discrete entropy is always non-negative. This article shows that negative entropy is a significant flaw when entropy is used as a sensitivity measure in global sensitivity analysis. Global sensitivity analysis based on differential entropy cannot have negative entropy, just as Sobol sensitivity analysis does not have negative variance. Entropy is similar to variance but does not have the same properties. An alternative sensitivity measure based on the approximation of the differential entropy using dome-shaped functionals with non-negative values is proposed in the article. Case studies have shown that new sensitivity measures lead to a rational structure of sensitivity indices with a significantly lower proportion of higher-order sensitivity indices compared to other types of distributional sensitivity analysis. In terms of the concept of sensitivity analysis, a decrease in variance to zero means a transition from the differential to discrete entropy. The form of this transition is an open question, which can be studied using other scientific disciplines. The search for new functionals for distributional sensitivity analysis is not closed, and other suitable sensitivity measures may be found.

## 1. Introduction

Sensitivity analysis (SA) based on entropy uses entropy to quantify uncertainty as Sobol SA [1,2] uses variance. Probability distributions with low variance have low entropy, while probability distributions with high variance have high entropy.

From a mathematical point of view, entropy is a certain additive functional on the probability distributions of possible states of a given system [3]. Entropy-based SA belongs to the category of distributional SA, which includes, for example, methods [4,5,6,7,8,9]. In these SAs, uncertainty is characterized by examining the entire distribution of model outputs, not just its variance.

There exist two popular indices based on entropy that have been used for SA. The first is entropy-based SA [10], which is based on the definition of Shannon’s entropy [11]. The second [12] is based on Kullback–Leibler entropy, which measures the difference between two probability distributions.

The use of entropy instead of variance is usually justified by the need to analyze the output random variable with heavy-tail or outliers [13]. SA based on entropy was used to study, for example, traffic flow [13], limit states of load-bearing structures [14,15], the seismic demand of concrete structures [16], and groundwater level [17].

Another group of tasks uses entropy to examine the state of a system in combination with certain types of SA, which may not be based on entropy. This group includes, for example, SA of the working process of heat exchangers [18], the hydraulic reliability of water distribution systems [19], shear stress distribution in a rectangular channel [20], creep of soft marine soil [21], air energy storage systems in coal-fired power plants [22], uncertainties of mathematical decision-making models [23,24,25,26,27], and many others.

Other types of SA include quantile-oriented methods [28,29,30,31,32,33], probability of failure [34,35,36,37,38,39,40,41], or decision-making approaches [42,43,44,45]. The subject of interest of these types of SA is the reliability of the system, which cannot be examined using SA distribution types.

The choice of SA method(s) remains an open question. A comparison of different types of sensitivity indices [39] shows that the sensitivity order from different types of SA can be the same or similar. Firm conclusions on the selection of the best SA method could not be reached in the majority of the studies pertaining to SA, which is understandable, because there are numerous ways to define which is the best one [46]. Sobol SA is very popular (see, e.g., [47,48,49,50,51,52,53,54]); on the contrary, less unified types of SA are less widespread [55,56,57,58,59,60,61,62,63,64]. In general, a global SA, which analyzes the influence of the variability of inputs throughout their distribution range and can describe the influence of interactions between input variables on the output, can be recommended.

This article aims to research global SA based on differential entropy using case studies. The concept of estimating sensitivity indices is described and the reasons why negative differential entropy is an undesirable part of SA are mentioned. The motivation for this work is to propose an alternative sensitivity measure based on the approximation of the differential entropy using functionals with non-negative values.

## 2. Entropy of a Random Variable

The concept of entropy for a discrete random variable was introduced by Claude Shannon [11] as a useful benchmark in information theory. The entropy of discrete random variable *Y* having a probability mass function can be written using the equation:

(1) $$H\left(Y\right)=-\sum_{i=1}^{n}P\left(y_{i}\right)\cdot \log_{b}\left(P\left(y_{i}\right)\right).$$

A valued characteristic of discrete entropy is that the entropy of a discrete random variable *Y* is zero or positive because the probabilities *P*(*y_i_*) in Equation (1) are in [0, 1]. This is also an important difference from differential entropy.

The differential (continuous) entropy can be defined using the following formula:

(2) $$H\left(R\right)=-\int_{-\infty }^{\infty }f\left(r\right)\log_{b}\left(f\left(r\right)\right)dr.$$

**Figure 1:**
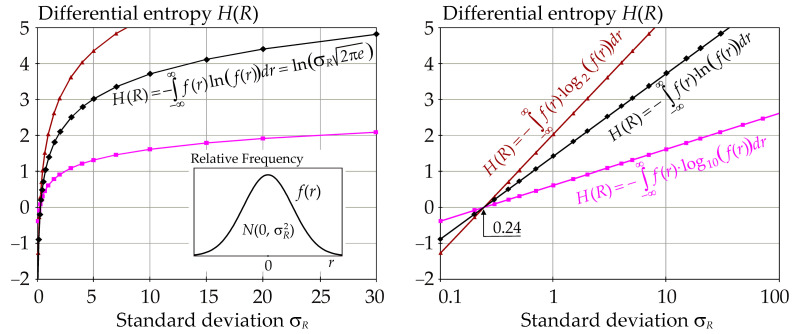
Differential entropy *H*(*R*) vs. *σ_R_* from Equation (2) using Gauss pdf of *f*(*r*).

*R* is a continuous random variable with the probability density function (pdf) *f*(*r*) on the real line. The differential entropy is not a limit of the Shannon entropy for *n* → ∞, although Equation (2) resembles an intuitive extension of Equation (1). In particular, it may be problematic that the differential entropy may be negative if *f*(*r*) > 1. For Gauss pdf, this occurs when the standard deviation *σ_R_* of *f*(*r*) is very small, which is illustrated by the example with mean value *μ_R_* = 0, where *σ_R_* is parameter of the graph—see Figure 1.

Entropy is a measure of uncertainty similar to variance. Higher entropy indicates higher uncertainty or higher variance. An important difference occurs with small uncertainties. If *R* has Gauss pdf, then the negative values of the differential entropy *H*(*R*) decrease when *σ_R_* decreases with the limit *H*(*R*) → −∞ when *σ_R_* → 0—see Figure 1. The same is true for other classic pdfs. Unlike the discrete case, the entropy of a continuous system does not remain invariant during the transformation of the coordinate systems [65].

The right part of Figure 1 shows that the dependence *H*(*R*) vs. ln(*σ_R_*) is linear, similarly, the dependence *H*(*R*) vs. ln(*σ_R_*·*σ_R_*) is also linear. It can be noted that the linear dependence of *H*(*R*) vs. ln(*σ_R_*) is observed for the Gauss pdf of *R* but does not occur generally for each pdf. For example, the dependence ln(*σ_R_*) vs. *H*(*R*) is linear if the variation coefficient is constant for log-normal pdf of *R*.

## 3. Entropy-Based Sensitivity Analysis

In SA, entropy is used as a measure of uncertainty for two types of sensitivity indices [10,12]. Both SA analyze changes in the probability density function (pdf) of the model output under the condition that one or more input random variables are fixed. The first concept of SA [10] is based on conditional entropy, which directly uses the definition of Shannon’s entropy. The second concept of SA [12] is based on the conditional relative entropy called Kullback–Leibler entropy.

This article builds on the first concept [10], but with the implementation of differential entropy according to Equation (2) and with the introduction of new alternative sensitivity measures.

Any computational model may be regarded as a function *R* = *g*(*X*), where *R* is a scalar model output, and *X* is a vector of *M* uncertain model inputs {*X*_1_, *X*_2_, … *X_M_*}, where statistical independence is assumed between inputs.

### 3.1. Sensitivity Indices based on Differential Entropy H(R)

Global sensitivity indices based on entropy can be formulated analogously to Sobol sensitivity indices [1,2] with the difference that variance is replaced by entropy [10]. Can global sensitivity indices be formulated using Equation (2), and with the shortcoming that the differential entropy can be negative when the variance is small? The answer can be obtained by analyzing the sensitivity index of the first and higher orders. The first-order entropy-base sensitivity index *T_i_* can be written as:

(3) $$T_{i}=\frac{H\left(R\right)-E\left(H\left(R|X_{i}\right)\right)}{H\left(R\right)},\;T_{i}\in \left[0,\;1\right],$$

where the mean value *E* [·] is taken across all likely values of *X_i_*. *H*(*R*|*X_i_*) is the conditional differential entropy, which represents the average loss of random variability on model output *R* when the input value of *X_i_* is known.

The values of *H*(*R*) and *H*(*R*|*X_i_*) must be such that *T_i_* ∈[0, 1]. In the limit case, if *R*|*X_i_* loses all random variability (*σ_R|Xi_* = 0), then the expected influence of *X_i_* on *R* is 100%, which means *T_i_* = 1. Therefore, *H*(*R*|*X_i_*) must be equal to zero and not −∞ as given by Equation (2)—see Figure 1. Equation (2) has the drawback that it can give negative entropy, which allows a sensitivity greater than 100%, *T_i_* > 1, which is not desired in the SA concept. On the other hand, Equation (2) satisfies the second extreme *H*(*R*) = *H*(*R*|*X_i_*), *T_i_* = 0, where fixing *X_i_* does not influence the pdf of output *R*. From the point of view of the SA concept, there are problematic cases where the variance of the output decreases to zero.

The variance and entropy of *R* further decrease if two or more input variables are fixed. The second-order entropy-base sensitivity index *T_ij_* is computed with the fixing of pairs *X_i_*, *X_j_*:

(4) $$T_{ij}=\frac{H\left(R\right)-E\left(H\left(R|X_{i},X_{j}\right)\right)}{H\left(R\right)}-T_{i}-T_{j},$$

where the mean value *E* [·] is taken across all likely values of *X_i_* and *X_j_*. The third-order sensitivity index, *E_ijk_*, is computed analogously:

(5) $$T_{ijk}=\frac{H\left(R\right)-E\left(H\left(R|X_{i},X_{j},X_{k}\right)\right)}{H\left(R\right)}-T_{i}-T_{j}-T_{k}-T_{ij}-T_{ik}-T_{jk}.$$

All input random variables are assumed to be statistically independent. The sum of all indices must be equal to one:

(6) $$\sum_{i}T_{i}+\sum_{i}\sum_{j>i}T_{ij}+\sum_{i}\sum_{j>i}\sum_{k>j}T_{ijk}+\ldots +T_{123\ldots M}=1.$$

The total entropy-base sensitivity index *T_Ti_* can be written as:

(7) $$T_{Ti}=1-\frac{H\left(R\right)-E\left(H\left(R|X_{\sim i}\right)\right)}{H\left(R\right)},$$

where the second term in the numerator contains the conditional entropy evaluated for the input random variable *X_i_* and fixed variables (*X*_1_, *X*_2_, …, *X_i−1_*, *X_i+1_*, …, *X_M_*).

The higher the order of the sensitivity index, the more input variables are fixed and the lower the entropy of the output *R*, including negative values. Each additional order of the sensitivity index has one additional random variable fixed until all inputs are fixed and the output becomes deterministic. During this process, both the entropy and the variance of *R* decrease. While the variance decreases to zero, the entropy decreases to negative values, which is particularly problematic when estimating higher-order sensitivity indices.

If all input random variables are fixed, then the output *R* is deterministic and the variance of the output *R* is zero. This occurs when the last-order sensitivity index is computed. Although the deterministic value of the output should have zero entropy according to Equation (1), the differential entropy according to Equation (2) extends to minus infinity. From the point of view of the SA concept, the entropy needs to decrease to zero when the variance of the output reaches zero.

This article seeks new forms of functionals as alternatives to Equation (2) rather than the application of Kullback–Leibler (K-L) (relative) entropy [66,67]. In terms of the concept of global SA, alternative forms of functional are sought so that the entropy is not negative when the variance of the output goes to zero.

### 3.2. Approximation of Differential Entropy by Functional $\tilde{H}\left(R\right)$ for Sensitivity Indices

From an SA point of view, Equation (2) is a functional that assigns a real value to function *f*(*r*). The modified Equation (2) should transform the inputs to the logarithm in the interval of zero to one so that the output value (entropy) cannot be negative for small *σ_R_* and also differ as little as possible from the differential entropy if *f*(*r*) < 1. The function should be increasing and decreasing approximately according to Equation (2) to fit Equation (2) well in the unproblematic areas. One such function, which is useful in modifying Equation (2), is the hyperbolic tangent:

(8) $$g\left(z\right)=\sqrt[{t}]{\tanh\left(z^{t}\right)},$$

where *z* = *f*(*r*).

**Figure 2:**
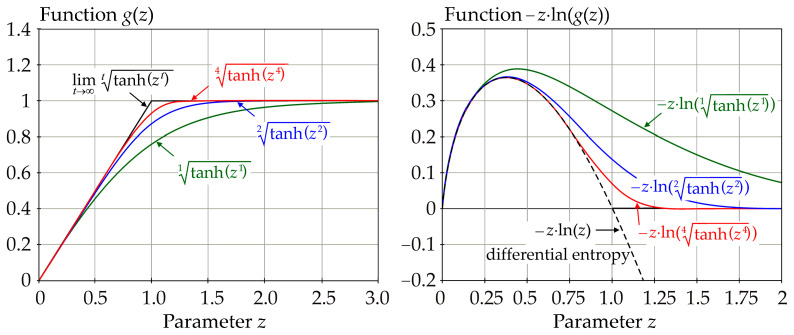
Examples of *g*(*z*) vs. *z* and examples of *z*·ln(*g*(*z*)) vs. *z* with using a natural logarithm.

In Equation (8), the larger the exponent *t*, the better *g*(z) fits the bilinear function—see left graph in Figure 2. Function *g*(*z*) does not provide an output greater than one, with the limit case in the form *g*(*z*) = *z*, *z*∈[0, 1); *g*(*z*) = 1, z∈[1, ∞]—see Figure 2.

Substituting Equation (8) into Equation (2), we obtain Equation (9) in the form:

(9) $$\tilde{H}\left(R\right)=-\int_{-\infty }^{\infty }f\left(r\right)\log_{b}\left(g\left[f\left(r\right)\right]\right)dr=-\int_{-\infty }^{\infty }f\left(r\right)\log_{b}\left(\sqrt[{t}]{\tanh\left({\left[f\left(r\right)\right]}^{t}\right)}\right)dr,$$

where $\tilde{H}\left(R\right)$ ≥ 0 and $\tilde{H}\left(R\right)$ → 0 if *σ_R_* → 0. The right side of Figure 2 shows examples of the plots of the natural logarithm functions that are used in Equation (9).

**Figure 3:**
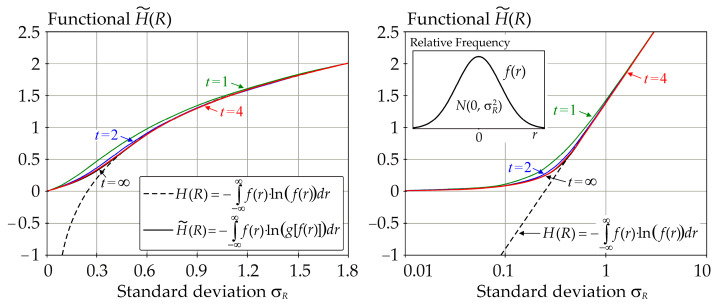
Approximation of *H*(*R*) by $\tilde{H}\left(R\right)$ using natural logarithm and Gauss pdf of *f*(*r*).

From the point of view of SA, Equation (9) is a functional that has the properties required in the decomposition of sensitivity indices. In the limit case *t* → ∞, the positive values of the logarithm are simply replaced by zero in the integral in Equation (9). It is apparent from the example shown in Figure 3 that the functional from Equation (9) differs from the differential entropy from Equation (2) only for small *σ_R_*; otherwise,$\tilde{H}\left(R\right)$ ≈ *H*(*R*) approximately according to Equation (2).

Equation (9) prevents the logarithm from returning a positive value within the integral when *f*(*r*) > 1. Let *f*(*r*) be a Gauss pdf and a natural logarithm is used in Equation (9). Then, the values of the functional $\tilde{H}\left(R\right)$ are plotted in Figure 3, including a comparison with the differential entropy *H*(*Y*) computed according to Equation (2). Equation (9) perfectly approximates the differential entropy from Equation (2) if large values of *σ_R_* are used. It can be noted that there is not much difference between the values plotted from Equation (9) for *t* = 4 (red curve) and *t* = ∞ (black curve)—see Figure 3.

The defect in Equation (2) could be engineered by using an additional condition, which replaces the negative entropy values with zero if such a situation occurs. However, this solution can assign zero differential entropy, even in the case where the random variable does not yet have zero standard deviation *σ_R_* > 0, so a gradual decrease to zero is more appropriate.

The sensitivity indices based on Equation (9) are computed according to Equations (3) to (7), with the difference that the differential entropy is replaced by the functional $\tilde{H}\left(R\right)$ according to Equation (9). Sensitivity indices based on $\tilde{H}\left(R\right)$ are denoted as ${\tilde{T}}_{i}$,${\tilde{T}}_{ij}$,${\tilde{T}}_{ijk}$, each index is in the interval [0, 1] and the sum of all sensitivity indices is equal to one.

### 3.3. Approximation of Differential Entropy by Functional $\hat{H}\left(R\right)$ and Sensitivity Indices

Let us consider another functional $\hat{H}\left(R\right)$ approximating Equation (2) such that $\tilde{H}\left(R\right)$ ≥ 0

(10) $$\hat{H}\left(R\right)=c_{1}\cdot \int_{-\infty }^{\infty }\sqrt{f\left(r\right)}\cdot e^{-2\cdot {\left(f\left(r\right)\right)}^{2}}dr,\;\mathrm{where}c_{1}=\frac{0.794423}{\ln\left(b\right)}$$

In order to approach Equation (2), *c*_1_ can be computed from the condition

(11) $$c_{1}\cdot \max\left(\sqrt{z}\cdot e^{-2\cdot z^{2}}\right)=\max\left(-z\cdot \log_{b}\left(z\right)\right),$$

where the maximum can be found in z∈[0, ∞) for the following arguments:

(12) $$z_{1}\underset{z}{=\mathrm{Argmax}}\;\left(\sqrt{z}\cdot e^{-2\cdot z^{2}}\right)=\frac{\sqrt{2}}{4},\;\;\;z_{2}\underset{z}{=\mathrm{Argmax}}\;\left(-z\cdot \log_{b}\left(z\right)\right)=\frac{1}{e}.$$

Upon substituting *z*_1_, *z*_2_ into (11), *c*_1_ can be computed as

(13) $$c_{1}=\frac{-\frac{1}{e}\cdot \log_{b}\left(\frac{1}{e}\right)}{\sqrt{\frac{\sqrt{2}}{4}}\cdot e^{-2\cdot {\left(\frac{\sqrt{2}}{4}\right)}^{2}}}=\frac{2^{\frac{3}{4}}\cdot e^{-\frac{3}{4}}}{\ln\left(b\right)}\approx \frac{0.794423}{\ln\left(b\right)}.$$

**Figure 4:**
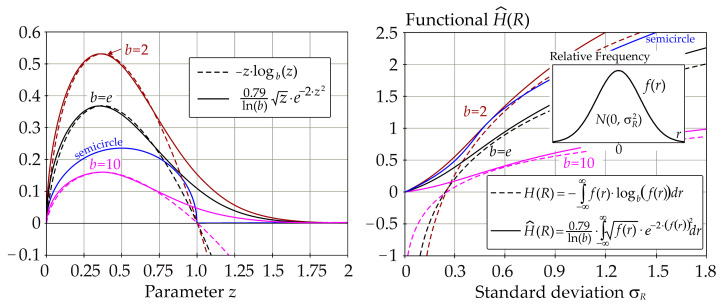
Approximation of *H*(*R*) by $\hat{H}\left(R\right)$ using three types of logarithm and Gauss pdf of *f*(*r*).

The left part of Figure 4 shows the variants of the functions that are integrated in Equation (10). Figure 4 shows that Equation (10) does not approximate the differential entropy as perfectly as Equation (9); however, this will not present a problem. The function used in Equation (10) differs more or less from the function *z*·log*_b_*(*z*) for both small and large values of *z* = *f*(*r*)—see the left part of Figure 4. Using the Gauss pdf of *f*(*r*), it is shown that large deviations in the approximation of the differential entropy are observed for both large and small values of *σ_R_*—see the right part of Figure 4.

Although the approximation of the differential entropy using Equation (10) is not as perfect as Equation (9), it is not a shortcoming in the evaluation of the sensitivity indices, as shown in the case studies below.

Sensitivity indices based on $\hat{H}\left(R\right)$ are computed according to Equation (3) to (7), with the difference that the differential entropy is replaced by the functional $\hat{H}\left(R\right)$ according to Equation (10). Sensitivity indices based on $\hat{H}\left(R\right)$ are denoted as ${\hat{T}}_{i}$,${\hat{T}}_{ij}$,${\hat{T}}_{ijk}$. The functional gives a non-negative output and is equal to zero when *σ_R_* = 0, each index is in the interval [0, 1] and the sum of all sensitivity indices is equal to one.

## 4. Standard Distribution-Based Sensitivity Analyzes

The sensitivity analysis described in the previous chapter is based on the probability distribution of all possible outcomes of the random phenomenon *R* being observed. This type of SA can be categorized as distribution-based SA, because the result of SA depends on the whole probability distribution of random variable *R* and not only on one moment, as is the case, for example, with Sobol SA. Other types of distribution-based sensitivity analysis that are relevant for comparison are Cramér-von Mises SA and Borgonovo moment-independent SA.

### 4.1. Cramér-von Mises Sensitivity Indices

Let Φ*_R_* be the distribution function of *R*, where *R* is a model output, and *X* is a vector of *M* uncertain model inputs {*X*_1_, *X*_2_, … *X_M_*} with the assumption of statistical independence.

(14) $$\Phi_{R}\left(p\right)=P\left(R\leq p\right)=E\left(1_{R\leq p}\right),\;\mathrm{for}\;p\in R,$$

Let $\Phi_{R}^{i}$ be the conditional distribution function of *R* conditionally on *X_i_*:

(15) $$\Phi_{R}^{i}\left(p\right)=P\left(R\leq p|X_{i}\right)=E\left(1_{R\leq p}|X_{i}\right),\;\mathrm{for}\;p\in R,$$

The first-order Cramér-von Mises index *G_i_* is determined by measuring the distance between probability Φ*_Z_*(*t*) and conditional probability $\Phi_{Z}^{i}$(*t*) when an input is fixed [68].

(16) $$G_{i}=\int_{-\infty }^{\infty }\frac{E\left[{\left(\Phi_{R}\left(p\right)-\Phi_{R}^{i}\left(p\right)\right)}^{2}\right]}{\Phi_{R}\left(p\right)\left(1-\Phi_{R}\left(p\right)\right)}d\Phi_{R}\left(p\right),$$

The second-order Cramér-von Mises index *G_ij_* can be written using [68] as

(17) $$G_{ij}=\int_{-\infty }^{\infty }\frac{E\left[{\left(\Phi_{R}\left(p\right)-\Phi_{R}^{ij}\left(p\right)\right)}^{2}\right]}{\Phi_{R}\left(p\right)\left(1-\Phi_{R}\left(p\right)\right)}d\Phi_{R}\left(p\right)-G_{i}-G_{j},$$

Since sensitivity indices *G_i_*, *G_ij_*, etc., are based on Hoeffding decomposition, the sum of all sensitivity indices is one [68]. Other characteristics of Cramér-von Mises indices and their behavior in engineering applications are mentioned in [39,69].

### 4.2. Borgonovo Moment-Independent Sensitivity Indices

Borgonovo moment-independent sensitivity indices [70] examine the whole distribution of inputs and outputs.

(18) $$B_{i}=\frac{1}{2}E\int_{-\infty }^{\infty }\left|f\left(r\right)-f_{R|X_{i}}\left(r\right)\right|dr,$$

where *f(r)* is the pdf of *R* and *f_R│__Xi_(r)* is the conditional pdf of *R* with fixed parameter *X_i_* [70]. 

Upon fixing pairs *X_i_*, *X_j_*, we obtain the second-order index *B_ij_*, where *i* < *j*. Upon fixing triplets *X_i_*, *X_j_*, *X_k_*, we obtain the third-order index *B_ijk_*, where *i* < *j* < *k*, etc. The higher the order of the index, the greater its value, and the index of the last order is equal to one, 0 ≤ *B_i_* ≤ *B_ij_* ≤ … ≤ *B*_1,2,…,*M*_ ≤ 1 [70]. Compared to SA, which has the sum of all indices equal to one, Borgonovo indices are less practical, especially the last index with fixed inputs, which is always equal to one and does not provide any new information. Identification of the influence of *X_i_* using total indices is not possible for Borgonovo indices. The advantage of Borgonovo indices is their evaluation of the whole distribution of the output in a more transparent way than presented by [68]—see Equation (18) vs. Equation (16). The second advantage is that the input variables can be statistically correlated, which is difficult to ensure for other types of indices based on decomposition, such as Sobol indices.

## 5. Variance-Based Sensitivity Analysis

Sobol SA [1,2] is a variance-based SA, which decomposes the variance of the model output into segments that can be attributed to inputs or sets of inputs. Sobol SA is a classical method, which is an important part of the research of computational models in stochastic structural mechanics—see, e.g., [71,72,73]. Although Sobol SA is of a different type than the SA in the previous chapters, supplementing the case study with Sobol indices is useful for comparison.

Although the differential entropy is dependent on the entire shape of the pdf, variance is an important characteristic for the computation of *H*(*R*). Entropy is a measure of system uncertainty similar to variance. Entropy increases when variance increases. Variance dependency makes Equation (3) similar to Sobol’s first-order sensitivity index:

(19) $$S_{i}=\frac{V\left(R\right)-E\left(V\left(R|X_{i}\right)\right)}{V\left(R\right)},$$

where *V*(*R*) is the variance and *V*(*R*|*X_i_*) is the conditional variance of the model output *R*. 

Higher-order Sobol sensitivity indices can be written analogously—see, e.g., [74].

## 6. The Case Studies

The resistance *R* of a steel member of a rectangular cross-section in tension is studied using six SA methods. Two new types of sensitivity indices, which are based on functionals $\tilde{H}\left(R\right)$ and $\hat{H}\left(R\right)$, are compared with four classical types of sensitivity indices in the case study.

### 6.1. Computational Model

The resistance *R* is the product of three random variables: yield strength *f_y_*, thickness *t*_2_, and width *b*. Statistical characteristics of *f_y_*, *t*_2_ and *b* are taken into consideration using the results of experimental research [75,76], where steel grade S355 was studied for selected steel products—see Table 1.

The cross-sectional area is expressed as the product of the thickness and width. The resistance *R* is the product of yield strength *f_y_* and cross-sectional area *t*_2_·*b*—see Equation (20).

(20) $$R=f_{y}\cdot t_{2}\cdot b.$$

The mean value *μ_R_* of the product *R* can be obtained from Equation (21):

(21) $$\mu_{R}=\mu_{fy}\cdot \mu_{t2}\cdot \mu_{b}.$$

The variance of the product *R* can be obtained from Equation (22):

(22) $$\sigma_{R}^{2}=\left(\mu_{b}^{2}+\sigma_{b}^{2}\right)\cdot \left(\mu_{t2}^{2}\cdot \sigma_{fy}^{2}+\sigma_{fy}^{2}\cdot \sigma_{t2}^{2}\right)+\mu_{fy}^{2}\cdot \left(\sigma_{t2}^{2}\cdot \left(\mu_{b}^{2}+\sigma_{b}^{2}\right)+\mu_{t2}^{2}\cdot \sigma_{b}^{2}\right),$$

where *σ_R_* is the standard deviation. 

Although all input random variables have Gauss pdf, their product has non-zero skewness *a_R_*—see Equation (23).

(23) $$a_{R}=6\cdot \frac{\mu_{R}}{\sigma_{R}^{3}}\cdot (\mu_{fy}^{2}\cdot \sigma_{t2}^{2}\cdot \sigma_{b}^{2}+\sigma_{fy}^{2}\cdot \mu_{t2}^{2}\cdot \sigma_{b}^{2}+\sigma_{fy}^{2}\cdot \sigma_{t2}^{2}\cdot \mu_{b}^{2}+4\cdot \sigma_{fy}^{2}\cdot \sigma_{t2}^{2}\cdot \sigma_{b}^{2}),$$

**Figure 5:**
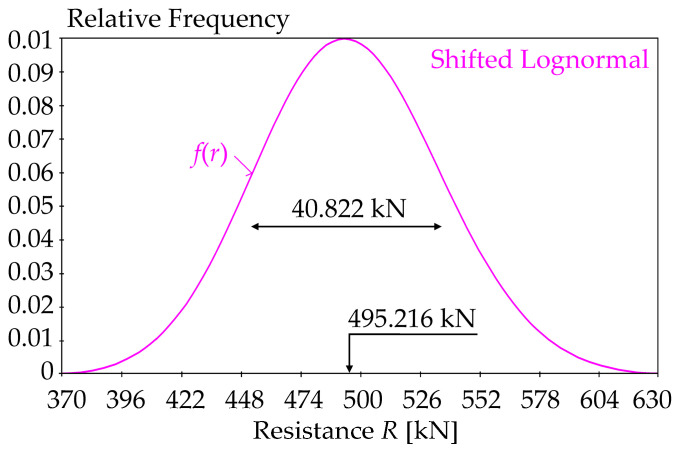
The probability density function of resistance *R* based on Table 1.

The following statistical characteristics of output *R*: *μ_R_ =* 495.216 kN, *σ_R_* = 40.822 kN, *a_R_ =* 0.11 are obtained upon substituting the statistical characteristics of the input random variables from Table 1—see Figure 5.

To evaluate global SA, the pdf of product *R* can be reliably approximated using a log-normal pdf with three parameters—*μ_R_*, *σ_R_*, and *a_R_* [69]. The three-parameter log-normal pdf can be used to estimate the sensitivity index, even if one of the three variables in Equation (20) is fixed as deterministic [32]. If two input variables are fixed, *a_R_* = 0 and product *R* has a Gauss pdf.

### 6.2. The Results of the Case Studies

**Figure 6:**
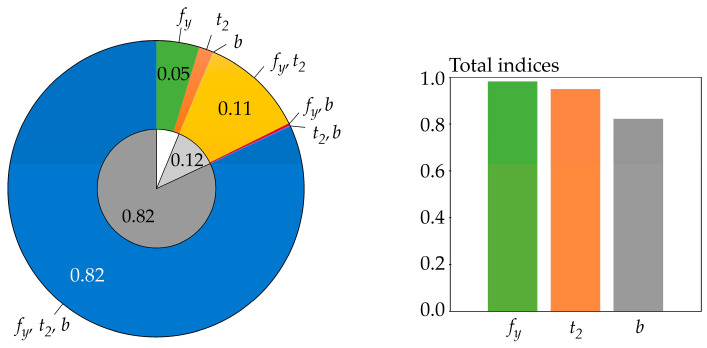
Sensitivity indices based on differential entropy *H*(*R*) with *b* = *e*.

**Figure 7:**
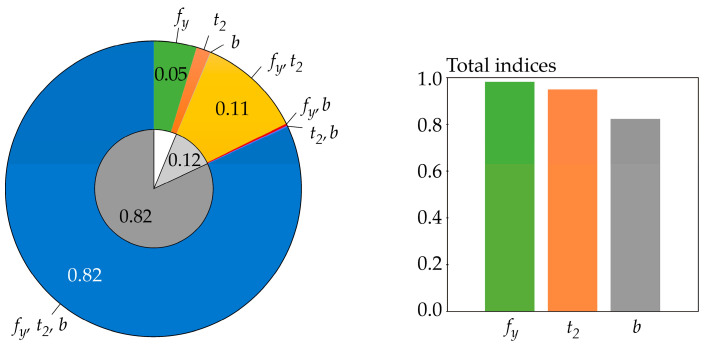
Sensitivity indices based on functional $\tilde{H}\left(R\right)$ with *b* = *e*; *t* = 4.

**Figure 8:**
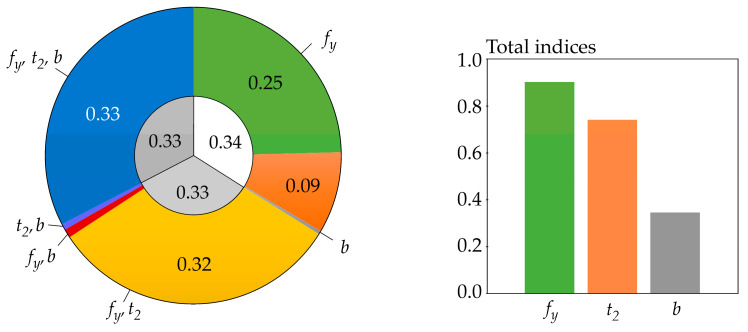
Sensitivity indices based on functional $\hat{H}\left(R\right)$ with *b* = *e*.

**Figure 9:**
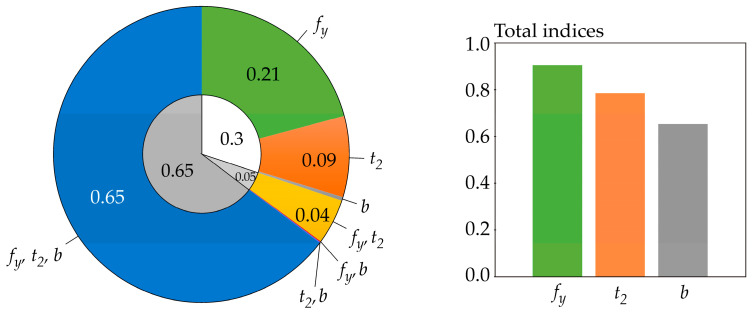
Gamboa sensitivity indices.

**Figure 10:**
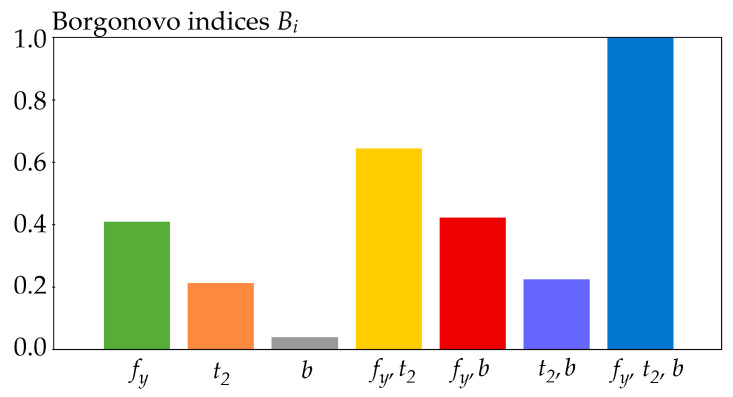
Borgonovo sensitivity indices.

**Figure 11:**
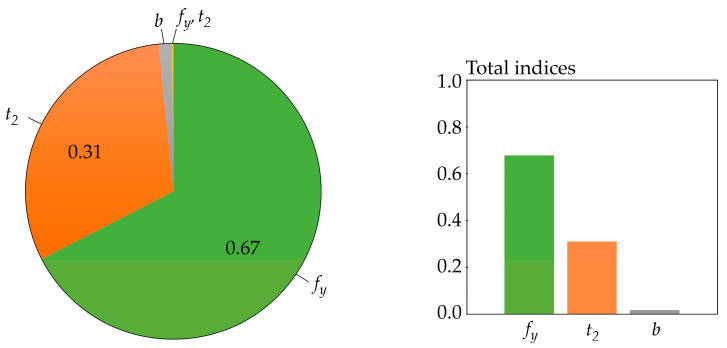
Sobol sensitivity indices.

The sensitivity indices are estimated using the Latin Hypercube Sampling method (LHS) [77,78] in combination with numerical integration. In equations where the arithmetic mean *E* (·) is used, its value is estimated using 1000 LHS runs. The three-parameter log-normal pdf of *f*(*r*) is used [69]. In all cases, integration is performed numerically by Simpson’s rule, using more than 10,000 integration steps over the interval [*μ_R_* − 10*σ_R_*, *μ_R_* + 10*σ_R_*]. If the lower bound of the domain of *f*(*r*) is greater than *μ_R_* − 10*σ_R_*, then integration is performed from the lower bound of *f*(*r*). Numerical integration is not used for Sobol sensitivity indices, which are computed analytically using Equation (22). Cramér-von Mises sensitivity indices are estimated using the algorithm described in [39,69], with the difference that three input random variables are used in this article. Borgonovo sensitivity indices were estimated according to the procedure in [39]. Further details of the numerical estimates of the sensitivity indices can be found in [39,69,72]. The results of sensitivity analyzes are shown in Figure 6, Figure 7, Figure 8, Figure 9, Figure 10 and Figure 11.

Sensitivity indices based on differential entropy *H*(*R*) are computed for *b* = *e*, but the same values can also be obtained for *b* = 2 or 10—see Figure 6. The sensitivity index of the last third-order was computed using the formal assumption that *H*(*R*|*X*_1_, *X*_2_, *X*_3_) = 0.

Sensitivity indices based on functional $\tilde{H}\left(R\right)$ are computed for *b* = *e* and *t* = 4, but practically the same values were obtained for *t* = 1, 2, 6—see Figure 7. The sensitivity index of the last third order was computed using $\tilde{H}$(*R*|*X*_1_, *X*_2_, *X*_3_) = 0.

The sensitivity indices based on functional $\hat{H}\left(R\right)$ are computed for *b* = *e*, but practically the same values were obtained for *b* = *2*, 10—see Figure 8. The sensitivity index of the last third-order was computed using $\hat{H}$(*R*|*X*_1_, *X*_2_, *X*_3_) = 0.

## 7. Discussion

**Figure 12:**
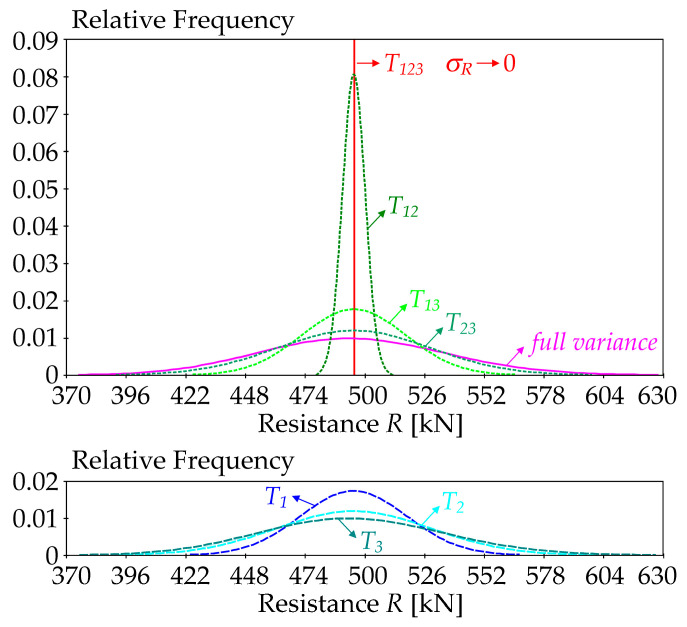
Probability density functions of resistance *R* with all combinations of input fixations.

The case study presented the results of several types of SA using the input random variables listed in Table 1, which are typical in structural mechanics. For SA based on the differential entropy, the values of relative frequency are especially important—see Figure 12. The combination of fixed and random input variables changes the variance of output variable *R*, with the peak of the pdf changing from 0.01 to infinity—see Figure 12. Fixing all three inputs leads to zero variance of *R* and theoretically infinite pdf value—see the red line in Figure 12. The pdf of *R* with all random inputs (full variance) is depicted in pink—see Figure 5. Figure 12 shows the pdf’s, which have the inputs fixed at the mean values.

Zero entropy replaces infinity when estimating the sensitivity index of the last third-order—see the red line in Figure 12. The sum of all sensitivity indices is equal to one only if *E*(*H*(*R*|*X*_1_, *X*_2_, *X*_3_)) = 0—see Equation (5). This means that the entropy of the sensitivity index of the last order (deterministic variables) must be calculated according to Equation (1).

In terms of the concept of sensitivity analysis, differential and discrete entropy are two related concepts, where the decrease of variance to zero (occurring gradually by fixing the input variables in all combinations) means a transition from differential to discrete entropy. The study suggests that global sensitivity analysis can help elucidate the nature of the transition between differential and discrete entropy.

The results depicted in Figure 6 and Figure 7 are practically the same because the estimates of the sensitivity indices in both cases are obtained using *f*(*r*) < 0.08 (relatively small values), i.e., in the region where $\tilde{H}\left(R\right)$ is very precisely equal to *H*(*R*). Although the values of *f*(*r*) are relatively small, using the differential entropy without further modifications does not provide a sufficiently general solution. The use of $\tilde{H}\left(R\right)$ provides a more general possibility of attaining *f*(*r*) > 1, even during the computation of sensitivity indices of lower orders, not just the last one.

All of the utilized SA types identically identified the sensitivity of the output *R* to the inputs in the following descending order: *f_y_*, *t*_2_, *b*. This sensitivity order was determined using total indices, except for Borgonovo SA, where total indices do not exist. The large value of the sensitivity index of the last order causes the difference between the total indices of certain SA types to be very small—see Figure 6 and Figure 7. In contrast, Sobol SA (Figure 11) and SA based on $\hat{H}\left(R\right)$—see Figure 8—which clearly identify a strong influence of *f_y_*, provide clear identification of the influential and non-influential inputs.

Although the sensitivity order is the same, the sizes of sensitivity indices of the same order based on $\hat{H}\left(R\right)$ differ from the sizes of indices based on *H*(*R*) or $\tilde{H}\left(R\right)$. The results of SA based on $\hat{H}\left(R\right)$ have a smaller value of the index of the last (third) order, which does not provide any new useful information for determining the sensitivity order of input variables. Gamboa SA also has a large share of the sensitivity index of the last order. Borgonovo SA has a last-order sensitivity index value implicitly equal to one. With a bit of exaggeration, the sensitivity index of the last order can be described as a “ballast” index, which does not provide useful information for determining the sensitivity order, either directly or using total indices.

The largest sum of first-order sensitivity indices (sum of all *S_i_* is 0.998) and very small higher-order sensitivity indices is given by Sobol SA—see Figure 11. This has also been observed for other tasks [31,47,71]. If the sum of all *S_i_* is equal to one, then the sensitivity order can be determined using only the *S_i_* indices, which are the same as the total indices, and higher order sensitivity indices do not have to be calculated. Easy interpretation of SA results, often carried out only with *S_i_*, is one of the features that makes Sobol SA popular.

A relatively large sum of all first-order sensitivity indices (0.34) was also obtained using SA based on $\hat{H}\left(R\right)$—see Figure 8. Gamboa sensitivity indices have the sum of all first-order sensitivity indices equal to 0.3; however, the last-order sensitivity index has a relatively high value of 0.65—see Figure 9.

New distribution-oriented sensitivity indices, which are an alternative to other types of distribution SA such as Cramér-von Mises SA [68] or Borgonovo moment-independent SA [70], have been proposed using functional $\hat{H}\left(R\right)$. The case study showed that the sensitivity indices based on functional $\hat{H}\left(R\right)$ have a good structure, which provides clear information about the sensitivity order of input variables—see Figure 8. The properties of these indices are mainly influenced by the beginnings of the curves integrated in Equation (10) and are shown on the left side of Figure 4. Virtually the same indices were obtained when the curves integrated in Equation (10) were replaced by a semicircle for *z* ∈ [0, 1] or zero for *z* > 1—see the blue curve on the left side of Figure 4.

In addition to the semicircle, it is also possible to experiment with other dome-shaped curves, which can suitably replace the function integrated in Equation (10). Further qualitative research could study the influence of dome shapes on the structure of sensitivity indices to find suitable curves for specific types of tasks. In general, it is possible to aim to maximize the share of first- and low-order sensitivity indices similar to Sobol’s indices. The results can be used in the application of acquired knowledge in understanding similar cases. Based on the findings from the case studies, new functionals with properties usable in SA can be sought.

## 8. Conclusions

The presented article compared several types of sensitivity analyzes and presented new distribution-oriented sensitivity indices, which were formulated based on differential entropy research. The comparative studies have shown the rationality of new sensitivity measures, their advantages, and disadvantages, in contrast to other types of SA.

The search for functionals suitable for distributional sensitivity analysis (SA) is not closed and other suitable sensitivity measures can be found. Sensitivity measures reflecting clear contrasts between sensitivity indices with the clear identification of influential and non-influential input variables ought to be sought. Preference may be given to a large proportion of sensitivity indices of the first and lower orders, similar to Sobol SA.

Entropy is an alternative measure for variance, but other similar measures are possible. From the point of view of the SA concept, a decrease in variance to zero means a transition from the differential to discrete entropy; differential entropy alone is not enough. The basic ways of making this transition were formulated in this article. Further research may proceed with the analysis of the links between differential and discrete entropy in specific applications of sensitivity analysis.

## Figures and Tables

**Table 1:** Input random variables.

Characteristic	Index	Symbol	Mean Value *μ*	Standard Deviation σ
Yield Strength	1	*f_y_*	412.68 MPa	27.941 MPa
Thickness	2	*t*_2_	12 mm	0.55 mm
Width	3	*b*	100 mm	1 mm

^1^ All inputs have Gauss pdf and are uncorrelated.

## Data Availability

Not applicable.
